# Ergometrinine

**DOI:** 10.1107/S1600536810030825

**Published:** 2010-08-11

**Authors:** Stefan Merkel, Robert Köppen, Matthias Koch, Franziska Emmerling, Irene Nehls

**Affiliations:** a BAM Federal Institute for Materials Research and Testing, Department Analytical Chemistry, Reference Materials, Richard-Willstätter-Strasse 11, D-12489 Berlin-Adlershof, Germany

## Abstract

The absolute configuration of ergometrinine, C_19_H_23_N_3_O_2_ {systematic name: (6a*R*,9*S*)-*N*-[(*S*)-1-hy­droxy­propan-2-yl]-7-methyl-4,6,6a,7,8,9-hexa­hydro­indolo[4,3-*fg*]quinoline-9-carb­ox­amide}, was established based on epimerization reaction of ergometrine, which was followed by preparative HPLC. The non-aromatic ring (ring *C* of the ergoline skeleton) directly fused to the aromatic rings is nearly planar [maximum deviation = 0.271 (3) Å] and shows an envelope conformation, whereas ring *D*, involved in an intra­molecular N—H⋯N hydrogen bond, exibits a slightly distorted chair conformation. The structure displays undulating layers in the *ac* plane formed by O—H⋯O and N—H⋯O hydrogen bonds.

## Related literature

Ergometrinine is one of the main ergot alkaloids produced by the fungus *Claviceps purpurea* on cereal grains in the field, see: Crews *et al.* (2009 ▶); Müller *et al.* (2009 ▶). For investigations of the biologically inactive C8-(*S*)-isomer of ergometrinine, see: Pierri *et al.* (1982 ▶); Komarova & Tolkachev (2001 ▶). For the crystal structure of ergometrine maleate, see: Cejka *et al.* (1996 ▶).
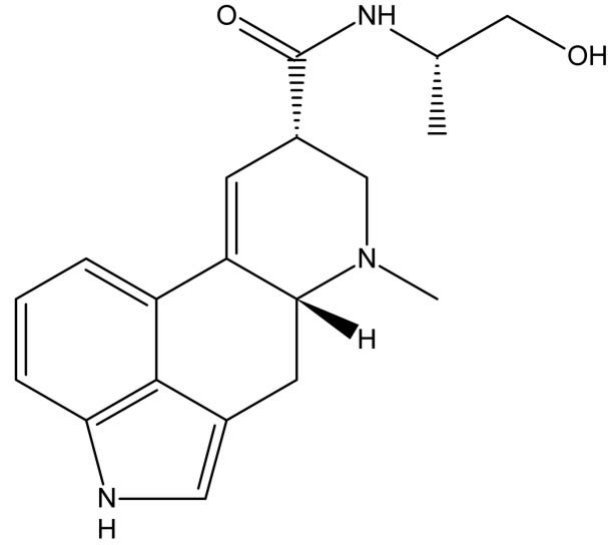


## Experimental

### 

#### Crystal data

C_19_H_23_N_3_O_2_
*M* *_r_* = 325.40
Orthorhombic, 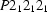

*a* = 7.4097 (5) Å
*b* = 12.7313 (7) Å
*c* = 18.2648 (9) Å
*V* = 1723.01 (17) Å^3^
*Z* = 4
Cu *K*α radiation
μ = 0.66 mm^−1^
*T* = 298 K
0.20 × 0.05 × 0.02 mm

#### Data collection

Enraf–Nonius CAD-4 diffractometer
Absorption correction: ψ scan (*CORINC*; Dräger & Gattow, 1971 ▶) *T*_min_ = 0.879, *T*_max_ = 0.986
4023 measured reflections
1889 independent reflections
1269 reflections with *I* > 2σ(*I*)
*R*_int_ = 0.056
3 standard reflections every 60 min  intensity decay: 2%

#### Refinement

*R*[*F* ^2^ > 2σ(*F* ^2^)] = 0.044
*wR*(*F* ^2^) = 0.112
*S* = 1.00
1889 reflections
219 parameters
H-atom parameters constrained
Δρ_max_ = 0.14 e Å^−3^
Δρ_min_ = −0.16 e Å^−3^

### 

Data collection: *CAD-4 Software* (Enraf–Nonius, 1989 ▶); cell refinement: *CAD-4 Software*; data reduction: *CORINC* (Dräger & Gattow, 1971 ▶); program(s) used to solve structure: *SIR97* (Altomare *et al.*, 1999 ▶); program(s) used to refine structure: *SHELXL97* (Sheldrick, 2008 ▶); molecular graphics: *ORTEPIII* (Burnett & Johnson, 1996 ▶); software used to prepare material for publication: *PLATON* (Spek, 2009 ▶).

## Supplementary Material

Crystal structure: contains datablocks I, global. DOI: 10.1107/S1600536810030825/sj5027sup1.cif

Structure factors: contains datablocks I. DOI: 10.1107/S1600536810030825/sj5027Isup2.hkl

Additional supplementary materials:  crystallographic information; 3D view; checkCIF report

## Figures and Tables

**Table 1:** Hydrogen-bond geometry (Å, °)

*D*—H⋯*A*	*D*—H	H⋯*A*	*D*⋯*A*	*D*—H⋯*A*
N1—H1*N*⋯N2	0.97	2.10	2.890 (4)	138
O2—H2*O*⋯O1^i^	1.01	1.68	2.684 (3)	172
N3—H3*N*⋯O2^ii^	0.95	1.97	2.918 (4)	173

Symmetry codes: (i) 
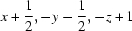
; (ii) 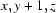.

**Fig. 1.**
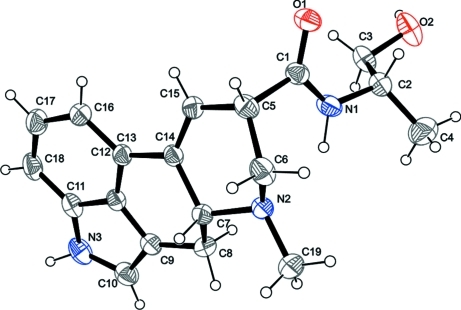
: ORTEP representation of the title compound with atomic labeling shown with 30% probability displacement ellipsoids.

**Fig. 2.**
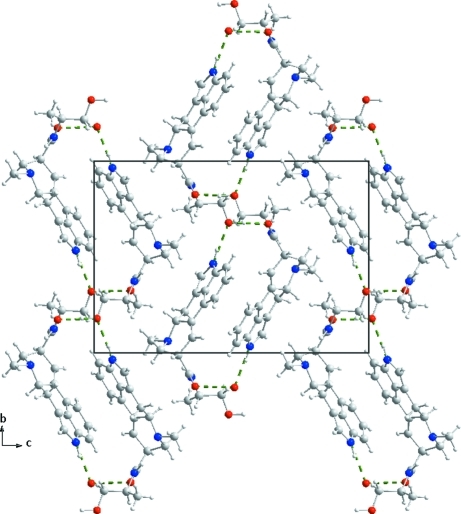
: View of the unit cell of the title compound along [100], showing the hydrogen-bonded layers stacked along the [001] direction. Hydrogen bonds are drawn as dashed lines.

## supplementary crystallographic information

### Comment

Ergometrinine is one of the main ergot alkaloids produced by the fungus *Claviceps purpurea* on cereal grains in the field. Contamination of flour and cereal based foods with ergot alkaloids including ergometrinine has previously been reported (Crews *et al.*, 2009, Müller  *et al.*, 2009). The biologically inactive C8-(*S*)-isomer ergometrinine (Pierri  *et al.*, 1982) can be converted to the biologically active C8-(*R*)-isomer ergometrine and vice versa (Komarova & Tolkachev, 2001). The molecule crystallizes in the orthorhombic space group *P*2_1_2_1_2_1_. The molecular structure of the compound and the atom labeling scheme are shown in Fig 1. The absolute configuration could not be defined confidently based on the single crystal diffraction data. It was however established based on epimerization reaction of ergometrine, whose absolute configuration was determined previously (Cejka *et al.*, 1996). Besides the intramolecular hydrogen bonds between N1-H1N and N2 (not shown in Fig. 2), each molecule is connected to four adjacent molecules via intermolecular hydrogen bonds (see dashed green bonds in Fig. 2). As a result undulating layers are formed in the the *ac* plane.

### Experimental

Ergometrine maleate salt was purchased from Sigma-Aldrich (Taufkirchen, Germany). The isomeric purity (> 99%) of ergometrine was proved by HPLC-FLD. The stereoselective conversion of ergometrine to ergometrinine was carried out as follows: 40 mg of ergometrine maleate were placed in a 250 ml round-bottom flask, dissolved in 100 ml methanol and 4 ml 28% ammonium hydroxide solution was added. The resulting mixture was stored at 40 °C in a drying cabinet in darkness for epimerization reaction. After 4 days liquid chromatography showed two different compounds, the reactant ergometrine and the product ergometrinine. Afterwards the solvent was removed *in vacuo* and the residue of compounds was redissolved in a mixture of water and acetonitrile (70:30, v:v) and subjected to preparative HPLC for separation and purification of the two epimers. Colourless crystals of ergometrinine were grown by slow solvent evaporation from acetonitrile:water (80:20, v:v) in the absence of light at ambient temperature.

### Refinement

In the absence of significant anomalous dispersion effects, Friedel pairs were merged.

The N—H and O—H hydrogen atoms were located in difference maps and and fixed in their found positions (AFIX 3) with *U*_iso_(H) = 1.2 of the parent atom *U*_eq_ or 1.5 *U*_eq_(C_methyl_, O).

### Crystal data

**Table:**

C_19_H_23_N_3_O_2_	*F*(000) = 696
*M**_r_* = 325.40	*D*_x_ = 1.254 Mg m^−^^3^
Orthorhombic, *P*2_1_2_1_2_1_	Cu *K*α radiation, λ = 1.54178 Å
Hall symbol: P 2ac 2ab	Cell parameters from 25 reflections
*a* = 7.4097 (5) Å	θ = 15–23°
*b* = 12.7313 (7) Å	µ = 0.66 mm^−^^1^
*c* = 18.2648 (9) Å	*T* = 298 K
*V* = 1723.01 (17) Å^3^	Needle, yellow
*Z* = 4	0.20 × 0.05 × 0.02 mm

### Data collection

**Table:**

Enraf–Nonius CAD-4 diffractometer	1269 reflections with *I* > 2σ(*I*)
Radiation source: rotating anode	*R*_int_ = 0.056
graphite	θ_max_ = 70.0°, θ_min_ = 4.2°
ω/2θ scans	*h* = −8→9
Absorption correction: ψ scan (CORINC; Dräger & Gattow, 1971)	*k* = −15→15
*T*_min_ = 0.879, *T*_max_ = 0.986	*l* = −22→22
4023 measured reflections	3 standard reflections every 60 min
1889 independent reflections	intensity decay: 2%

### Refinement

**Table:**

Refinement on *F*^2^	Primary atom site location: structure-invariant direct methods
Least-squares matrix: full	Secondary atom site location: difference Fourier map
*R*[*F*^2^ > 2σ(*F*^2^)] = 0.044	Hydrogen site location: inferred from neighbouring sites
*wR*(*F*^2^) = 0.112	H-atom parameters constrained
*S* = 1.00	*w* = 1/[σ^2^(*F*_o_^2^) + (0.0561*P*)^2^ + 0.0234*P*] where *P* = (*F*_o_^2^ + 2*F*_c_^2^)/3
1889 reflections	(Δ/σ)_max_ < 0.001
219 parameters	Δρ_max_ = 0.14 e Å^−^^3^
0 restraints	Δρ_min_ = −0.16 e Å^−^^3^

### Special details

**Table:**

Geometry. All e.s.d.'s (except the e.s.d. in the dihedral angle between two l.s. planes) are estimated using the full covariance matrix. The cell e.s.d.'s are taken into account individually in the estimation of e.s.d.'s in distances, angles and torsion angles; correlations between e.s.d.'s in cell parameters are only used when they are defined by crystal symmetry. An approximate (isotropic) treatment of cell e.s.d.'s is used for estimating e.s.d.'s involving l.s. planes.
Refinement. Refinement of *F*^2^ against ALL reflections. The weighted *R*-factor *wR* and goodness of fit *S* are based on *F*^2^, conventional *R*-factors *R* are based on *F*, with *F* set to zero for negative *F*^2^. The threshold expression of *F*^2^ > σ(*F*^2^) is used only for calculating *R*-factors(gt) *etc*. and is not relevant to the choice of reflections for refinement. *R*-factors based on *F*^2^ are statistically about twice as large as those based on *F*, and *R*- factors based on ALL data will be even larger.

### Fractional atomic coordinates and isotropic or equivalent isotropic displacement parameters (Å^2^)

**Table:**

	*x*	*y*	*z*	*U*_iso_*/*U*_eq_	
O1	0.2918 (3)	−0.17372 (18)	0.36716 (13)	0.0586 (7)	
O2	0.7137 (5)	−0.32052 (18)	0.48938 (13)	0.0815 (10)	
H2O	0.7329	−0.3191	0.5439	0.122*	
N1	0.5819 (4)	−0.1255 (2)	0.35374 (16)	0.0550 (8)	
H1N	0.6603	−0.0793	0.3259	0.066*	
N2	0.6383 (4)	0.06231 (19)	0.26795 (14)	0.0519 (8)	
N3	0.7306 (5)	0.4652 (2)	0.43312 (17)	0.0666 (9)	
H3N	0.7302	0.5372	0.4476	0.080*	
C1	0.4047 (6)	−0.1103 (2)	0.34594 (17)	0.0464 (9)	
C2	0.6592 (5)	−0.2226 (3)	0.38200 (17)	0.0520 (9)	
H2	0.5736	−0.2793	0.3715	0.062*	
C3	0.6825 (6)	−0.2176 (2)	0.46383 (19)	0.0602 (11)	
H2A	0.7839	−0.1727	0.4761	0.072*	
H2B	0.5749	−0.1889	0.4864	0.072*	
C4	0.8315 (6)	−0.2470 (4)	0.3419 (2)	0.0890 (15)	
H4A	0.8070	−0.2530	0.2904	0.133*	
H4B	0.8802	−0.3121	0.3596	0.133*	
H4C	0.9170	−0.1916	0.3500	0.133*	
C5	0.3492 (5)	−0.0110 (2)	0.30590 (17)	0.0493 (9)	
H5	0.2276	−0.0228	0.2865	0.059*	
C6	0.4692 (6)	0.0159 (3)	0.24169 (19)	0.0584 (11)	
H6A	0.4950	−0.0471	0.2137	0.070*	
H6B	0.4077	0.0653	0.2098	0.070*	
C7	0.6047 (5)	0.1676 (2)	0.29911 (16)	0.0440 (8)	
H7	0.5654	0.2129	0.2588	0.053*	
C8	0.7810 (5)	0.2148 (3)	0.32985 (19)	0.0527 (9)	
H8A	0.8389	0.1644	0.3620	0.063*	
H8B	0.8631	0.2303	0.2899	0.063*	
C9	0.7408 (5)	0.3130 (2)	0.37121 (18)	0.0491 (9)	
C10	0.8341 (6)	0.4010 (3)	0.3898 (2)	0.0622 (10)	
H10	0.9516	0.4157	0.3752	0.075*	
C11	0.5652 (6)	0.4189 (3)	0.44279 (19)	0.0543 (10)	
C12	0.5701 (5)	0.3240 (2)	0.40378 (16)	0.0435 (8)	
C13	0.4242 (5)	0.2560 (2)	0.40020 (16)	0.0436 (8)	
C14	0.4491 (5)	0.1623 (2)	0.35402 (16)	0.0417 (8)	
C15	0.3382 (5)	0.0807 (2)	0.35753 (18)	0.0459 (8)	
H15	0.2495	0.0802	0.3936	0.055*	
C16	0.2697 (5)	0.2850 (3)	0.43839 (18)	0.0545 (9)	
H16	0.1681	0.2421	0.4373	0.065*	
C17	0.2672 (7)	0.3792 (3)	0.4786 (2)	0.0685 (12)	
H17	0.1631	0.3962	0.5045	0.082*	
C18	0.4104 (7)	0.4473 (3)	0.4817 (2)	0.0656 (11)	
H18	0.4047	0.5094	0.5083	0.079*	
C19	0.7721 (7)	0.0655 (3)	0.2083 (2)	0.0793 (14)	
H19A	0.7780	−0.0020	0.1850	0.119*	
H19B	0.8884	0.0830	0.2279	0.119*	
H19C	0.7371	0.1175	0.1730	0.119*	

### Atomic displacement parameters (Å^2^)

**Table:**

	*U*^11^	*U*^22^	*U*^33^	*U*^12^	*U*^13^	*U*^23^
O1	0.0606 (17)	0.0495 (13)	0.0658 (15)	−0.0125 (13)	0.0060 (14)	0.0047 (12)
O2	0.129 (3)	0.0458 (13)	0.0695 (17)	0.0021 (17)	−0.0340 (18)	0.0006 (12)
N1	0.048 (2)	0.0480 (16)	0.0684 (19)	−0.0048 (15)	−0.0015 (17)	0.0171 (14)
N2	0.067 (2)	0.0411 (14)	0.0477 (15)	0.0007 (15)	0.0157 (16)	−0.0004 (12)
N3	0.090 (3)	0.0410 (15)	0.0693 (19)	−0.0083 (18)	−0.017 (2)	−0.0049 (15)
C1	0.054 (3)	0.0427 (17)	0.0426 (17)	−0.0070 (19)	0.0016 (18)	−0.0021 (14)
C2	0.054 (2)	0.0444 (18)	0.058 (2)	−0.0008 (18)	−0.0048 (19)	0.0014 (16)
C3	0.071 (3)	0.0408 (18)	0.069 (2)	−0.0040 (19)	−0.017 (2)	0.0001 (16)
C4	0.069 (3)	0.094 (3)	0.104 (4)	0.013 (3)	0.018 (3)	0.011 (3)
C5	0.051 (2)	0.0449 (17)	0.0523 (19)	−0.0076 (18)	−0.0095 (18)	0.0020 (15)
C6	0.084 (3)	0.0473 (19)	0.0442 (18)	0.000 (2)	−0.005 (2)	−0.0034 (16)
C7	0.050 (2)	0.0414 (15)	0.0408 (16)	0.0026 (17)	0.0039 (17)	0.0042 (14)
C8	0.046 (2)	0.0538 (19)	0.058 (2)	−0.0014 (18)	0.0112 (18)	0.0019 (16)
C9	0.051 (2)	0.0431 (18)	0.0531 (19)	−0.0019 (17)	−0.0028 (19)	0.0044 (15)
C10	0.059 (3)	0.054 (2)	0.074 (2)	−0.007 (2)	−0.008 (2)	0.0077 (19)
C11	0.072 (3)	0.0394 (17)	0.0520 (19)	0.0029 (19)	−0.010 (2)	0.0001 (16)
C12	0.052 (2)	0.0381 (16)	0.0408 (17)	0.0039 (18)	−0.0029 (17)	0.0020 (14)
C13	0.049 (2)	0.0412 (16)	0.0409 (16)	0.0084 (18)	0.0016 (17)	0.0048 (13)
C14	0.048 (2)	0.0368 (15)	0.0406 (16)	0.0042 (16)	−0.0001 (16)	0.0035 (13)
C15	0.046 (2)	0.0443 (16)	0.0476 (17)	0.0042 (17)	0.0016 (17)	0.0024 (14)
C16	0.058 (3)	0.0512 (18)	0.0541 (19)	0.0053 (19)	0.010 (2)	0.0019 (17)
C17	0.087 (3)	0.060 (2)	0.059 (2)	0.021 (3)	0.018 (2)	−0.0003 (19)
C18	0.098 (3)	0.0418 (18)	0.057 (2)	0.018 (2)	0.004 (2)	−0.0066 (16)
C19	0.104 (4)	0.061 (2)	0.073 (2)	0.004 (3)	0.042 (3)	−0.0040 (19)

### Geometric parameters (Å, °)

**Table:**

O1—C1	1.226 (4)	C6—H6B	0.9700
O2—C3	1.410 (4)	C7—C14	1.530 (4)
O2—H2O	1.0068	C7—C8	1.544 (5)
N1—C1	1.334 (5)	C7—H7	0.9800
N1—C2	1.457 (4)	C8—C9	1.490 (5)
N1—H1N	0.9707	C8—H8A	0.9700
N2—C6	1.466 (5)	C8—H8B	0.9700
N2—C19	1.474 (5)	C9—C10	1.360 (5)
N2—C7	1.477 (4)	C9—C12	1.405 (5)
N3—C11	1.371 (5)	C10—H10	0.9300
N3—C10	1.372 (5)	C11—C18	1.397 (6)
N3—H3N	0.9537	C11—C12	1.403 (4)
C1—C5	1.517 (4)	C12—C13	1.386 (5)
C2—C4	1.505 (5)	C13—C16	1.391 (5)
C2—C3	1.506 (5)	C13—C14	1.473 (4)
C2—H2	0.9800	C14—C15	1.325 (4)
C3—H2A	0.9700	C15—H15	0.9300
C3—H2B	0.9700	C16—C17	1.407 (5)
C4—H4A	0.9600	C16—H16	0.9300
C4—H4B	0.9600	C17—C18	1.371 (6)
C4—H4C	0.9600	C17—H17	0.9300
C5—C15	1.503 (4)	C18—H18	0.9300
C5—C6	1.511 (5)	C19—H19A	0.9600
C5—H5	0.9800	C19—H19B	0.9600
C6—H6A	0.9700	C19—H19C	0.9600
			
C3—O2—H2O	109.5	N2—C7—H7	107.2
C1—N1—C2	123.2 (3)	C14—C7—H7	107.2
C1—N1—H1N	116.4	C8—C7—H7	107.2
C2—N1—H1N	117.7	C9—C8—C7	110.0 (3)
C6—N2—C19	110.1 (3)	C9—C8—H8A	109.7
C6—N2—C7	110.3 (3)	C7—C8—H8A	109.7
C19—N2—C7	111.9 (3)	C9—C8—H8B	109.7
C11—N3—C10	108.5 (3)	C7—C8—H8B	109.7
C11—N3—H3N	112.1	H8A—C8—H8B	108.2
C10—N3—H3N	137.2	C10—C9—C12	105.6 (3)
O1—C1—N1	122.8 (3)	C10—C9—C8	135.7 (4)
O1—C1—C5	121.1 (3)	C12—C9—C8	118.6 (3)
N1—C1—C5	116.1 (3)	C9—C10—N3	110.5 (4)
N1—C2—C4	109.6 (3)	C9—C10—H10	124.7
N1—C2—C3	111.1 (3)	N3—C10—H10	124.7
C4—C2—C3	113.3 (4)	N3—C11—C18	133.5 (3)
N1—C2—H2	107.5	N3—C11—C12	106.4 (3)
C4—C2—H2	107.5	C18—C11—C12	120.1 (4)
C3—C2—H2	107.5	C13—C12—C11	122.8 (3)
O2—C3—C2	107.9 (3)	C13—C12—C9	128.3 (3)
O2—C3—H2A	110.1	C11—C12—C9	108.9 (3)
C2—C3—H2A	110.1	C12—C13—C16	116.9 (3)
O2—C3—H2B	110.1	C12—C13—C14	115.8 (3)
C2—C3—H2B	110.1	C16—C13—C14	127.2 (3)
H2A—C3—H2B	108.4	C15—C14—C13	122.0 (3)
C2—C4—H4A	109.5	C15—C14—C7	122.3 (3)
C2—C4—H4B	109.5	C13—C14—C7	115.7 (3)
H4A—C4—H4B	109.5	C14—C15—C5	123.0 (3)
C2—C4—H4C	109.5	C14—C15—H15	118.5
H4A—C4—H4C	109.5	C5—C15—H15	118.5
H4B—C4—H4C	109.5	C13—C16—C17	119.9 (4)
C15—C5—C6	110.0 (3)	C13—C16—H16	120.0
C15—C5—C1	111.1 (2)	C17—C16—H16	120.0
C6—C5—C1	113.8 (3)	C18—C17—C16	123.4 (4)
C15—C5—H5	107.2	C18—C17—H17	118.3
C6—C5—H5	107.2	C16—C17—H17	118.3
C1—C5—H5	107.2	C17—C18—C11	116.8 (3)
N2—C6—C5	109.9 (3)	C17—C18—H18	121.6
N2—C6—H6A	109.7	C11—C18—H18	121.6
C5—C6—H6A	109.7	N2—C19—H19A	109.5
N2—C6—H6B	109.7	N2—C19—H19B	109.5
C5—C6—H6B	109.7	H19A—C19—H19B	109.5
H6A—C6—H6B	108.2	N2—C19—H19C	109.5
N2—C7—C14	109.8 (2)	H19A—C19—H19C	109.5
N2—C7—C8	110.5 (3)	H19B—C19—H19C	109.5
C14—C7—C8	114.6 (2)		
			
C2—N1—C1—O1	5.7 (5)	N3—C11—C12—C9	0.7 (4)
C2—N1—C1—C5	−171.7 (3)	C18—C11—C12—C9	−178.6 (3)
C1—N1—C2—C4	142.9 (4)	C10—C9—C12—C13	178.9 (3)
C1—N1—C2—C3	−91.1 (4)	C8—C9—C12—C13	−3.3 (5)
N1—C2—C3—O2	165.5 (3)	C10—C9—C12—C11	−1.0 (4)
C4—C2—C3—O2	−70.6 (4)	C8—C9—C12—C11	176.9 (3)
O1—C1—C5—C15	96.5 (4)	C11—C12—C13—C16	−0.9 (5)
N1—C1—C5—C15	−86.0 (4)	C9—C12—C13—C16	179.3 (3)
O1—C1—C5—C6	−138.6 (3)	C11—C12—C13—C14	177.2 (3)
N1—C1—C5—C6	38.8 (4)	C9—C12—C13—C14	−2.6 (5)
C19—N2—C6—C5	166.6 (3)	C12—C13—C14—C15	165.5 (3)
C7—N2—C6—C5	−69.4 (3)	C16—C13—C14—C15	−16.6 (5)
C15—C5—C6—N2	48.3 (4)	C12—C13—C14—C7	−17.5 (4)
C1—C5—C6—N2	−77.1 (3)	C16—C13—C14—C7	160.3 (3)
C6—N2—C7—C14	50.1 (3)	N2—C7—C14—C15	−14.9 (4)
C19—N2—C7—C14	173.1 (3)	C8—C7—C14—C15	−140.1 (3)
C6—N2—C7—C8	177.6 (3)	N2—C7—C14—C13	168.2 (3)
C19—N2—C7—C8	−59.4 (4)	C8—C7—C14—C13	43.0 (4)
N2—C7—C8—C9	−171.3 (2)	C13—C14—C15—C5	173.7 (3)
C14—C7—C8—C9	−46.5 (4)	C7—C14—C15—C5	−3.1 (5)
C7—C8—C9—C10	−155.5 (4)	C6—C5—C15—C14	−13.5 (5)
C7—C8—C9—C12	27.4 (4)	C1—C5—C15—C14	113.5 (4)
C12—C9—C10—N3	0.9 (4)	C12—C13—C16—C17	−0.5 (5)
C8—C9—C10—N3	−176.4 (4)	C14—C13—C16—C17	−178.3 (3)
C11—N3—C10—C9	−0.5 (4)	C13—C16—C17—C18	1.2 (5)
C10—N3—C11—C18	179.1 (4)	C16—C17—C18—C11	−0.6 (6)
C10—N3—C11—C12	−0.2 (4)	N3—C11—C18—C17	−179.9 (4)
N3—C11—C12—C13	−179.1 (3)	C12—C11—C18—C17	−0.7 (5)
C18—C11—C12—C13	1.5 (5)		

### Hydrogen-bond geometry (Å, °)

**Table:**

*D*—H···*A*	*D*—H	H···*A*	*D*···*A*	*D*—H···*A*
N1—H1N···N2	0.97	2.10	2.890 (4)	138.
O2—H2O···O1^i^	1.01	1.68	2.684 (3)	172.
N3—H3N···O2^ii^	0.95	1.97	2.918 (4)	173.

Symmetry codes: (i) *x*+1/2, −*y*−1/2, −*z*+1; (ii) *x*, *y*+1, *z*.
